# Trend of incompleteness of the race/color variable in hospitalizations due to COVID-19 whose outcome was death in Brazil, 2020–2022

**DOI:** 10.11606/s1518-8787.2024058006032

**Published:** 2024-08-23

**Authors:** Hebert Luan Pereira Campos dos Santos, Emmanuel Santos Trindade, Esly Rebeca Amaral Oliveira, Marcos Vinicius da Silva Cordeiro, Rian Silva de Oliveira, Elvira Caires de Lima, Adriano Maia dos Santos, Nília Maria de Brito Lima Prado

**Affiliations:** I Universidade Federal da Bahia Instituto de Saúde Coletiva Salvador BA Brasil Universidade Federal da Bahia. Instituto de Saúde Coletiva. Salvador, BA, Brasil; II Universidade Estadual do Sudoeste da Bahia Vitória da Conquista BA Brasil Universidade Estadual do Sudoeste da Bahia. Vitória da Conquista, BA, Brasil; III Universidade Federal da Bahia Instituto Multidisciplinar em Saúde Programa de Pós-Graduação em Psicologia da Saúde Vitória da Conquista BA Brasil Universidade Federal da Bahia. Instituto Multidisciplinar em Saúde. Programa de Pós-Graduação em Psicologia da Saúde. Vitória da Conquista, BA, Brasil; IV Universidade Federal do Espírito Santo Programa de Pós-Graduação em Saúde Coletiva Vitória ES Brasil Universidade Federal do Espírito Santo. Programa de Pós-Graduação em Saúde Coletiva. Vitória, ES, Brasil; V Universidade Federal da Bahia Instituto Multidisciplinar em Saúde Programa de Pós-Graduação em Saúde Coletiva Vitória da Conquista BA Brasil Universidade Federal da Bahia. Instituto Multidisciplinar em Saúde. Programa de Pós-Graduação em Saúde Coletiva. Vitória da Conquista, BA, Brasil

**Keywords:** Information Systems, Black People, Time Series Studies, Health Observatory, COVID-19, Health of Ethnic Minorities, Pandemics, Ethnic Inequality

## Abstract

**OBJECTIVE:**

To analyze the incompleteness and trend of incompleteness of the race/color variable in hospitalizations due to COVID-19 whose outcome was death, in Brazil, between April 2020 and April 2022.

**METHODS:**

Ecological time series study on the incompleteness of the race/color variable in hospitalizations due to COVID-19 whose outcome was death in Brazil, its macro-regions and Federative Units (FU), by joinpoint regression, calculation of Monthly Percent Change (MPC) and Average Monthly Percent Change (AMPC), based on data from the Hospital Information System of the Unified Health System (SIH/SUS).

**RESULTS:**

The incompleteness of the race/color variable in COVID-19 hospitalizations with a death outcome in Brazil was 25.85%, considered poor. All regions of the country had a poor degree of incompleteness, except for the South, which was considered regular. In the period analyzed, the joinpoint analysis revealed a stable trend in the incompleteness of the race/color variable in Brazil (AMPC = 0.54; 95%CI: -0.64 to 1.74; p = 0.37) and in the Southeast (AMPC = -0.61; 95%CI: -3.36 to 2.22; p = 0.67) and North (AMPC = 3.74; 95%CI: -0.14 to 7.78; p = 0.06) regions. The South (AMPC = 5.49; 95%CI: 2.94 to 8.11; p = 0.00002) and Northeast (AMP = 2.50; 95%CI: 0.77 to 4.25; p = 0.005) regions showed an increase in the incompleteness trend, while the Midwest (AMPC = -2.91 ; 95%CI: -5.26 to -0.51; p = 0.02) showed a downward trend.

**CONCLUSION:**

The proportion of poor completeness and the stable trend of incompleteness show that there was no improvement in the quality of filling in the race/color variable during the COVID-19 pandemic in Brazil, a fact that may have increased health inequalities for the black population and made it difficult to plan strategic actions for this population, considering the pandemic context. The results found reinforce the need to encourage discussion on the subject, given that the incompleteness of health information systems increases inequalities in access to health services and compromises the quality of health data.

## INTRODUCTION

The development of health policies in line with the reality of the population is directly related to access to quality information, the availability of which is fundamental for analyzing the health situation and making decisions in the context of health policies and care, with a view to reducing inequities and inequalities in health^1^. In Brazil, the current governance structure for health data and information in the Unified Health System (SUS) is considered a robust initiative as it includes a range of Health Information Systems (SIS), nationwide and with a large part of the data available via Internet access, led by the Ministry of Health^2,3^.

As a joint effort, given the existence of numerous information systems structured based on individualized strategies, integration, and interoperability between SIS^4^ has currently been proposed with a view to strengthening “the process of planning, operating, and controlling health services”^3^. One of the pillars of this process is reliability, which involves quality indicators such as coverage, degree of completeness, reliability and timeliness of these systems, consistency, and validity of the data, which is fundamental for drawing up and implementing policies aimed at improving the health of the population^1,5^.

Considering the dimension of data completeness in the SUS, several Brazilian studies^6^ have indicated a high degree of variation in the incompleteness of many variables. The race/color variable, for example, has achieved a low degree of completeness in a set of conditions and diseases^9,10^. In the context of the coronavirus disease 2019 (COVID-19) pandemic, for example, studies have pointed to a lack of data disaggregated by race/color, which has made it impossible to reliably verify the impacts of the pandemic on this population and has hindered the development of specific strategic plans^11^. Santos et al.^11^ pointed out that, at the beginning of the pandemic, of the 27 Federative Units (FU), only 19% disclosed data disaggregated by race/color for confirmed cases, hospitalized cases of severe acute respiratory syndrome due to COVID-19, and deaths due to COVID-19.

Although the biological concept of race has been overcome from a theoretical point of view, race/color is understood as a social construct that determines objective conditions of inequality in the conditions of life and death and institutionalized racism in practices and services in Brazil^12^. Despite the extensive experience with the implementation of SIS in Brazil, the race/color variable having been included in the SUS Hospital Information System (SIH/SUS) since 2008, the *Política Nacional de Saúde Integral da População Negra* (PNSIPN – National Policy on Comprehensive Health of the Black Population^13^ guiding the filling in of this variable and the obligation defined by Ordinance No. 344 of 2017^14^, there is still great variation in the degree of incompleteness.

The SUS Indicators Panel^15^ already signaled the need to effectively fill in the race/color item in health information forms and systems. However, there are few^16^ published studies analyzing the degree of incompleteness of the race/color variable in the context of the COVID-19 pandemic, according to data from the SUS information systems.

Thus, the objective of this study is to analyze the incompleteness and trend of incompleteness of the race/color variable in hospitalizations for COVID-19 whose outcome was death, in Brazil, between April 2020 and April 2022.

## METHODS

This is an ecological time-series study analyzing the trend of the incompleteness of the race/color variable in hospitalizations due to COVID-19 whose outcome was death, in Brazil, its macro-regions and states, between April 2020 and April 2022.

### Data Sources and Variables Selected

The information on mortality used in this study comes from hospital admissions of patients whose outcome was death and whose main diagnosis was coronavirus infection of unspecified location (ICD B34.2) or coronavirus as a cause of diseases classified in other chapters (ICD B97.2), between April 2020 and April 2022. The microdata was obtained from the SIH/SUS of the SUS Information Technology Department (Datasus) of the Ministry of Health^19^. The variables used were death by race/color, region, and UF.

The time frame chosen comprises the months of April 2020 and April 2022. The delimitation of this time horizon is justified because April 2020 was the month in which the Federal Public Defender’s Office (DPU) filed a lawsuit to force the collection of data on race/color from epidemiological surveillance systems for COVID-19^20^ and, in April 2022, the closure of the Public Health Emergency of National Importance (ESPIN) was declared^21^.

Filling in the race/color variable is governed by Ordinance No. 344/2017, which establishes that the collection of the color questionnaire and filling in the race/color field are mandatory for professionals working in health services, in order to respect the health user’s self-declaration criterion, within the standards used by the *Instituto Brasileiro de Geografia e Estatística* (IBGE – Brazilian Institute of Geography and Statistics) that appear on health information system forms as: 1) white; 2) black; 3) yellow (person of oriental origin: Japanese, Chinese, and Korean, among others); 4) brown (included in this category: brunette, mulatto, cabocla, cafuza, or any other mixed race of black person with person of another color or race); 5) indigenous (applies to indigenous people or Indians living in a village, as well as those who have declared themselves indigenous and live outside the village)^14^. The ordinance also establishes that, in the case of newborns, deaths, or situations in which the user is unable to self-declare, it will be up to family members or guardians to declare their color or ethnic-racial belonging and, in cases where there is no guardian, the health professionals who carry out the care must fill in the race/color field^14^.

### Data Analysis

The percentage of incompleteness (blank or ignored) of the race/color variable was calculated for Brazil, each geographic region (North, Northeast, South, Midwest, and Southeast), and UF, per month and for the entire period. Incompleteness refers to fields that are blank, not filled in, ignored, or situations in which it is filled in but with a non-existent/wrong number for the variable^22^. We used the cut-off points defined by Romero and Cunha^22^, widely used in the literature to define incompleteness: excellent, when the variable has less than 5% incompleteness; good (5.0% to 9.9% incompleteness); regular (10.0% to 19.9%); poor (20.0% to 49.9%); and very poor (50.0% or more incompleteness).

To analyze the trend of incompleteness of the race/color variable, joinpoint regression was used, using the Joinpoint Regression Program software version 4.8.0.1, assuming months of the year as the regressor variable, and stratification by region and state. Joinpoint regression is a way of analyzing temporal trends, evaluating joinpoints and whether there are changes in the pattern of this trend. This model adjusts a series of lines and their joinpoints on a logarithmic scale to demonstrate trends^23,24^.

The following were calculated: 1) the monthly percentage change (MPC) for each segment; and 2) the average monthly percentage change (AMPC), which is the weighted geometric average of the different MPCs, with a weight equal to the size of the segment for each time interval. The number of junction points to obtain the significant model was selected using the software’s default settings. For the significance test, the p-value was applied, which adjusts the best line for each segment. Once these segments have been established, the respective monthly percentages of change are estimated and tested. When there is a junction point where the direction is reversed or different trend patterns are observed, the periods are analyzed separately; if there is no change, the period is analyzed as a whole^23,24^.

MPC and AMPC associated with a p-value < 0.05 were considered statistically significant. The scenario of an increase in the trend of incompleteness indicated a worsening in the completion of the variable, and a decrease indicated an improvement in this scenario. The trend, when not significant (p-value ≥ 0.05), was considered stationary, i.e. it did not show growth or a statistically significant reduction in its time series, regardless of the MPC or AMPC value.

By using only anonymous and publicly available data, in accordance with Resolution 466 of 2012 of the National Health Council, it was not necessary to submit the project to an ethics committee for research with human beings.

## RESULTS

Between April 2020 and April 2022, 384,704 hospitalizations were recorded in Brazil due to COVID-19 resulting in death, 25.85% (n = 99,465) of which had incomplete completion of the race/color variable. Considering the entire period analyzed, incompleteness for the race/color variable in Brazil was considered poor (25.85%) according to the classification system proposed by Romero and Cunha^22^, a behavior similar to that of its regions, with the exception of the South, where it was considered regular (12.94%) (Table 1). Of the 27 Brazilian states, the majority (n = 11) had a poor degree of incompleteness, according to Table 1. The Figure illustrates the month-to-month variation in the incompleteness of the race/color variable in hospitalizations for COVID-19 whose outcome was death in Brazil and its regions. The joinpoint analysis reports that Brazil showed a trend towards stability in the incompleteness of the race/color variable in COVID-19 hospitalizations (AMPC = 0.54; 95%CI: -0.64 to 1.74; p = 0.37) (Table 2).

**Table 1:** Percentage of incompleteness of the race/color variable in hospitalizations due to COVID-19 whose outcome was death by geographic region and Federative Unit, Brazil, April/2020–April/2022.

Region/UF	Incompleteness (%)	Classification^a^
Brazil	25.85	Poor
Northern Region	20.72	Poor
Rondônia	34.52	Poor
Acre	10.59	Regular
Amazonas	15.77	Regular
Roraima	6.46	Good
Pará	21.13	Poor
Amapá	57.52	Very poor
Tocantins	3.01	Excellent
Northeast Region	39.18	Poor
Maranhão	51.76	Very poor
Piauí	50.36	Very poor
Ceará	27.93	Poor
Rio Grande do Norte	47.36	Poor
Paraíba	41.81	Poor
Pernambuco	36.78	Poor
Alagoas	28.28	Poor
Sergipe	75.67	Very poor
Bahia	32.65	Poor
Midwest Region	40.62	Poor
Mato Grosso do Sul	16.87	Regular
Mato Grosso	29.89	Poor
Goiás	51.12	Very poor
Federal District	57.28	Very poor
Southeast Region	22.17	Poor
Minas Gerais	16.6	Regular
Espírito Santo	18.27	Regular
Rio de Janeiro	38.72	Poor
São Paulo	18.39	Regular
Southern Region	12.94	Regular
Paraná	15.24	Regular
Rio Grande do Sul	16.32	Regular
Santa Catarina	2.72	Excellent

Source: Based on data from *Departamento de Informática do SUS* (Datasus – SUS Information Technology Department).

^a^ According to Romero and Cunha^22^.

**Figure 1:**
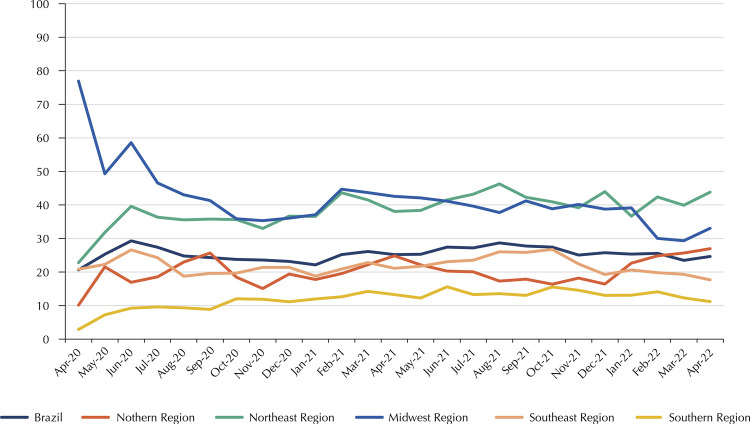
Percentage of incompleteness of the race/color variable in hospitalizations for COVID-19 whose outcome was death by geographic region and Federative Unit, Brazil, April/2020–April/2022.

**Table 2:** Trend of incompleteness of the race/color variable in hospitalizations due to COVID-19 whose outcome was death by geographic region and Federative Unita, Brazil, April/2020–April/2022.

Region/UF	Period	MPC (95%CI)	p-value	Trends	AMPC (95%CI)	p-value	Trends
Brazil	Apr/20–Jun/20	16.96 (4.92 to 30.38)	0.008	Growth	0.54 (-0.64 to 1.74)	0.37	Stability
	Jun/20–Nov/20	-4.66 (-7.89 to -1.33)	0.010	Reduction			
	Nov/20–Aug/21	2.48 (1.27 to 3.70)	0.0006	Growth			
	Aug/21–Apr/21	-2.04 (-3.19 to -0.87)	0.002	Reduction			
Northern Region	Apr/20–Jun/20	32.26 (-11.54 to 97.74)	0.16	Stability	3.74 (-0.14 to 7.78)	0.06	Stability
	Jun/20–Dec/21	-0.66 (-2.05 to 0.75)	0.33	Stability			
	Dec/21–Apr/22	11.71 (-1.63 to 26.86)	0.08	Stability			
Amazonas	Apr/20–Apr/22	-0.03 (-2.64 to 2.66)	0.98	Stability	-0.03 (-2.64 to 2.66)	0.98	Stability
Pará	Apr/20–Sep/20	13.17 (-3.99 to 33.41)	0.13	Stability	2.13 (-2.58 to 7.06)	0.38	Stability
	Sep/20–Aug/21	-10.31 (-15.30 to -5.02)	0.0009	Reduction			
	Aug/21–Apr/22	14.50 (5.67 to 24.07)	0.002	Growth			
Northeast Region	Apr/20–Jun/20	24.07 (0.54 to 53.09)	0.04	Growth	2.50 (0.77 to 4.25)	0.005	Growth
	Jun/20–Abr/22	0.73 (0.23 to 1.24)	0.006	Growth			
Maranhão	Apr/20–Jun/20	55.78 (-2.86 to 149.81)	0.06	Stability	2.06 (-3.19 to 7.60)	0.45	Stability
	Jun/20–Feb/22	0.47 (-0.92 to 1.89)	0.49	Stability			
	Feb/22–Abr/22	-21.77 (-51.22 to 25.45)	0.29	Stability			
Piauí	Apr/20–Jun/20	-31.85 (-55.81 to 5.10)	0.08	Stability	-3.09 (-6.43 to 0.37)	0.08	Stability
	Jun/20–Abr/22	0.06 (-0.96 to 1.10)	0.9	Stability			
Ceará	Apr/20–Sep/21	3.85 (1.65 to 6.09)	0.002	Growth	2.76 (-4.96 to 11.11)	0.49	Stability
	Sep/21–Dec/21	-28.11 (-60.93 to 32.29)	0.27	Stability			
	Dec/21–Apr/22	28.47 (5.94 to 55.80)	0.01	Growth			
Rio Grande do Norte	Apr/20–Apr/22	1.85 (0.64 to 3.07)	0.004	Growth	1.85 (0.64 to 3.07)	0.004	Growth
Paraíba	Apr/20–Apr/22	-0.39 (-2.23 to 1.47)	0.66	Stability	-0.39 (-2.23 to 1.47)	0.66	Stability
Pernambuco	Apr/20–Jun/20	38.54 (-3.12 to 98.10)	0.07	Stability	3.62 (-1.86 to 9.41)	0.2	Stability
	Jun/20–Nov/20	-9.16 (-18.87 to 1.72)	0.09	Stability			
	Nov/20–Feb/21	27.52 (-10.82 to 82.34)	0.17	Stability			
	Feb/21–Abr/22	-0.33 (-1.99 to 1.35)	0.68	Stability			
Alagoas	Apr/20–Aug/20	-36.29 (-57.68 to -4.08)	0.03	Reduction	-4.51 (-10.95 to 2.40)	0.2	Stability
	Aug/20–Abr/22	3.54 (-0.07 to 7.28)	0.05	Stability			
Bahia	Apr/20–Apr/22	-1.98 (-3.49 to -0.45)	0.01	Reduction	-1.98 (-3.49 to -0.45)	0.01	Reduction
Midwest Region	Apr/20–Nov/20	-9.27 (-12.53 to -5.90)	0.00003	Reduction	-2.91 (-5.26 to -0.51)	0.02	Reduction
	Nov/20–Feb/21	7.80 (-5.98 to 23.60)	0.26	Stability			
	Feb/21–Abr/22	-2.49 (-3.88 to -1.08)	0.002	Reduction			
Goiás	Apr/20–Apr/22	-1.79 (-2.95 to -0.62)	0.004	Reduction	-1.79 (-2.95 to -0.62)	0.004	Reduction
Federal District	Apr/20–Apr/22	-2.03 (-2.80 to -1.25)	0.00002	Reduction	-2.03 (-2.80 to -1.25)	0.00002	Reduction
Southeast Region	Apr/20–Jun/20	12.48 (-7.30 to 36.48)	0.21	Stability	-0.61 (-3.36 to 2.22)	0.67	Stability
	Jun/20–Sept/20	-10.06 (-25.88 to 9.14)	0.26	Stability			
	Sep/20–Sep/21	2.47 (1.14 to 3.82)	0.001	Growth			
	Sep/21–Apr/22	-4.97 (-7.40 to -2.48)	0.0008	Reduction			
Rio de Janeiro	Apr/20–Apr/22	0.65 (-0.48 to 1.80)	0.25	Stability	0.65 (-0.48 to 1.80)	0.25	Stability
São Paulo	Apr/20–Jun/20	17.57 (-11.27 to 55.79)	0.24	Stability	-1.77 (-5.74 to 2.36)	0.4	Stability
	Jun/20–Sept/20	-13.87 (-35.00 to 14.13)	0.27	Stability			
	Sep/20–Nov/21	0.90 (-0.58 to 2.40)	0.22	Stability			
	Nov/21–Apr/22	-8.23 (-13.83 to -2.27)	0.01	Reduction			
Southern Region	Apr/20–Jun/20	73.07 (32.04 to 126.84)	0.0005	Growth	5.49 (2.94 to 8.11)	0.00002	Growth
	Jun/20–Out/21	2.77 (1.60 to 3.95)	0.0001	Growth			
	Oct/21–Apr/22	-4.09 (-8.38 to 0.39)	0.07	Stability			
Paraná	Apr/20–Aug/20	31.79 (14.08 to 52.25)	0.0007	Growth	5.48 (2.92 to 8.11)	0.00002	Growth
	Aug/20–Abr/22	0.89 (-0.37 to 2.16)	0.16	Stability			

MPC: Monthly percentage change; 95%CI: 95% confidence interval; AMPC: Average monthly percent change.

^a^ The states of Rondônia, Acre, Roraima, Amapá, Tocantis, Minas Gerais, Sergipe, Espírito Santo, Minas Gerais, Rio Grande do Sul, Santa Catarina had at least one month with incompleteness equal to 0%, incompatible with the method based on logarithmic calculations, so they were excluded from the table.

In the North, incompleteness was considered poor. Of the seven states in the region, two (Rondônia and Pará) had a poor degree of incompleteness and Amapá was considered very poor (Table 1). In the joinpoint analysis, the region (AMPC = 3.74; 95%CI: -0.14 to 7.78; p = 0.06) and the states of Amazonas (AMPC = -0.03; 95%CI: -2.64 to 2.66; p = 0.98) and Pará (AMPC = 2.13; 95%CI: -2.58 to 7.06; p = 0.38) also tended towards stability (Table 2).

The Northeast had a poor degree of incompleteness (39.18%): six states were considered poor (Ceará, Rio Grande do Norte, Paraíba, Pernambuco, Alagoas, and Bahia) and three very poor (Maranhão, Piauí, and Sergipe) (Table 1). In the joinpoint analysis, the Northeast showed an upward trend (AMPC = 2.50; 95%CI: 0.77 to 4.25; p = 0.005), although most of the Northeastern states showed a stable trend, with the exception of Rio Grande do Norte, with an upward trend (AMPC = 1.85; 95%CI: 0.64 to 3.07; p = 0.004), and Bahia, with a downward trend (AMPC = -1.98; 95%CI: -3.49 to -0.45; p = 0.01) (Table 2).

The Midwest was the region with the highest degree of incompleteness (40.62%) and was classified as poor. Two states were considered very poor (Goiás and the Federal District) (Table 1). In the incompleteness trend analysis, the Midwest Region (AMPC: -2.91; 95%CI: -5.26 to -0.51; p = 0.02) tended to reduce, a behavior also shown by Goiás (AMPC = -1.79; 95%CI: -2.95 to -0.62; p = 0.004) and the Federal District (AMPC = -2.03; 95%CI: -2.80 to -1.25; p = 0.00002) (Table 2).

In the Southeast, the degree of incompleteness was considered poor (22.17%), although most states (São Paulo, Espírito Santo, and Minas Gerais) were considered regular (Table 1). In the joinpoint analysis, the Southeast (AMPC = -0.61;95%CI: -3.36 to 2.22; p = 0.67) and the states of Rio de Janeiro (AMPC = 0.65; 95%CI: -0.48 to 1.80; p = 0.25) and São Paulo (APMC = -1.77; 95%CI: -5.74 to 2.36]; p = 0.40) showed a stable trend (Table 2).

The South was the only region with a regular degree of incompleteness (12.94%) and the state of Santa Catarina was the only one in the country with an excellent degree of incompleteness (Table 1). In the joinpoint analysis, the region (AMPC = 5.49; 95%CI: 2.94 to 8.11; p = 0.00002) and the state of Paraná (AMPC = 5.48; 95%CI: 2.92 to 8.11; p = 0.00002) showed an upward trend (Table 2).

## DISCUSSION

The results of this research show that the degree of incompleteness of the race/color variable in hospitalizations for COVID-19 whose outcome was death, between April 2020 and April 2022, was poor throughout Brazil. These data corroborate the national literature regarding the degree of completion of the race/color variable in various SUS information systems, in different periods^8^ and in a set of diseases^16^.

On the other hand, the historical series revealed a trend of stability in the degree of incompleteness in the country, a result that diverges from that found by some national studies in some diseases and conditions^16^. These findings may be divergent, because in contexts of public health emergencies and high demand for health services, the availability and quality of data on race/color are often impaired^25^. Throughout the pandemic, despite some initiatives to improve data collection, difficulties in handling information systems have prevailed at all levels of management, either due to incomplete information, with varying quality between the agencies and institutions responsible for collection, or to changes in the collection process, flow, and instruments aimed at reporting cases^26^.

In the international context, for example, evidence has pointed to disparities between racial/ethnic groups in hospitalization and mortality rates, access to health services during the pandemic and vulnerability to COVID-19 being masked by racial/ethnic labels, indicating deficiencies in public health strategies and the need for systematic collection of these variables to identify risk groups^27^. It is known that many low- and middle-income countries do not have adequate surveillance systems and responsive health infrastructures to solve these problems reliably^30^. In this regard, Otu et al.^31^ recommend the creation of SIS that facilitate the acquisition and use of disaggregated data during pandemic contexts and beyond, emphasizing the possibility of identifying trends related to race/color and broader socioeconomic determinants, seeking to institute rapid policy changes based on intersectional approaches.

In this study, the South Region had the lowest average incompleteness of race/color records of hospitalizations due to COVID-19 whose outcome was death, a result also found by Souza et al.^16^, when they evaluated the incompleteness of the race/color records of the most prevalent diseases and conditions in the black population in the SIS between 2009 and 2018. Although there has been significant progress in the SIS in Brazil, certain problems and difficulties persist in some regions of the country, such as the North and Northeast^32^. Senna^33^ points out that this reflects the social and regional inequalities that exist in the country, which involve problems such as medical provision in certain locations and in places that are difficult to access, difficulties in retaining professionals, especially trained coders, among others.

Although Brazil has stood out in the formulation and evolution of the SIS in America^32^ and the percentage of incompleteness of the race/color field at the national level has decreased in the last 15 years^25^, the challenges for the qualification of data on the health of the black population still remain. Regarding race/color, various studies^6^ have pointed to the permanence of interpersonal, structural, and institutional racism in the collection of this variable by health service professionals. Geraldo et al.^34^, when describing and analyzing the implementation of the collection of race/color by professionals in the patient registration sector of a university hospital in the municipality of São Paulo, during the COVID-19 pandemic, found that most of the race/color records in the observed institution are made by heteroidentification, even though Ordinance No. 344/2017^14^ determines that filling in the field must respect the criterion of self-declaration by the health user, except in some scenarios.

Being asked which race/color the individual identifies with still makes many health professionals uncomfortable^34,35^, with a probable tendency towards whitening in heteroidentification. In addition to this, there is a lack of knowledge about the PNSIPN, little understanding of the importance of collecting the race/color questionnaire for the development of public policies on the part of health professionals, a lack of specific training for completing the race/color questionnaire, and the absence of audit projects to monitor its completion^7^. These aspects should be considered important barriers in the process of collecting and systematizing data, since they are what guide the formulation of health policies in the country.

One of the strategic actions of the III PNSIPN Operational Plan of 2017^36^ was to improve the collection, processing, analysis, and publication of data disaggregated by race/color. However, in Brazil, studies on the COVID-19 pandemic have pointed to an explicit policy of making data that reveal ethnic-racial inequalities invisible, especially due to the lack of disclosure of data disaggregated by race/color/ethnicity^11,22^. Even though it has been compulsory to fill in race/color since 2017^14^ , the variable was not included in the epidemiological bulletins published at the beginning of the pandemic in the country, and was only incorporated after pressure from social movements and entities linked to the field of health^11,36^. Araújo et al.^37^ pointed out, for example, that even when the Brazilian state began to disclose disaggregated data, it was of poor quality, a factor that made robust analysis of the pandemic’s impact on ethnic minorities impossible.

It should be recognized that the quality of information on race/color is a necessary condition for recognizing the impact of health inequities on mortality and access to health services. There is a need to improve the recording, collection, and dissemination of data in the context of epidemics such as that represented by COVID-19, considering not only race/color but also occupation, place of residence, and other variables that make it possible to understand the relationships between the process of social determination of health and its impact on the morbidity and mortality indicators of the Brazilian population^26^.

Regional discrepancies in the degree of completeness reveal important challenges for the Ministry of Health in implementing an SIS that effectively contributes to an equitable and universal SUS. Several factors may be involved in these regional disparities, such as: illegible entries, poor flow of information, lack of knowledge, or little importance on the part of professionals when filling in the data, among others^10^.

Some studies^10,22^, when analyzing Brazilian SIS, pointed to a higher degree of incompleteness in the initial years of analysis, with improvement over the years analyzed. However, in the context of health emergencies, robust SIS, considering coverage, degree of completeness, reliability, consistency, and validity of the data, are essential for the correct response to these events. Efforts to develop intersectoral policies to overcome the serious consequences of incomplete data must be undertaken in order to reduce social inequalities, especially for the most vulnerable populations.

This study has limitations regarding the methods, considering that the use of secondary data can lead to underestimation due to incompleteness and inconsistencies in the source databases. These limitations can be classified into three types, namely: the first concerns the extraction of death data from consolidated information on hospital admission authorizations (AIH); however, this is the only public domain SIH in the country that has made available data disaggregated by race/color, in public access, considering the updated codes of the International Classification of Diseases (ICD-10) for COVID-19. The second concerns the evaluation of the system’s coverage, which only covers hospitalizations carried out in the public network and those affiliated with the SUS, excluding hospitalizations carried out privately and by health plans. However, the SIH/SUS is an important database on hospital morbidity in Brazil and consolidates a large part of hospital admissions in the country. The third involves the fact that only the deaths of hospitalized patients who had an AIH were analyzed, excluding individuals who died without being hospitalized.

The results obtained point to some directions in the political-institutional context: the first refers to the need to strengthen the monitoring and evaluation of the actions proposed by the PNSIPN, since, even 15 years after its publication, central elements of the policy still remain unfulfilled, such as the permanent education of health workers on the importance of correctly filling in the race/color questionnaire for the SIS and for the PNSIPN to be effective. The second concerns the strengthening of SIS in the Brazilian context, since robust public health systems require consistent and reliable SIS. The third refers to the urgent need to strengthen the Brazilian health system’s preparation for and response to public health emergencies, with a view to developing efficient, effective, equitable actions that are in line with local realities, with emphasis on social inequalities, as well as differences in risk related to diseases, since up-to-date data can translate into better local and regional decision-making.

The challenges posed by the COVID-19 pandemic in countries like Brazil, marked by racism and significant social and racial inequalities, could mean an opportunity to redefine public health policies and the care offered to socially marginalized populations, driven by managers, public policy makers, professionals, and people committed to reducing racial inequalities in the country.
