# Supplementary material for: Trend of incompleteness of the race/color variable in hospitalizations due to COVID-19 whose outcome was death in Brazil, 2020–2022
Source: Rev Saude Publica. 2024 Aug 23;58:37. doi: 10.11606/s1518-8787.2024058006032 (PMC11548909; doi:10.11606/s1518-8787.2024058006032)
Supplement: Translation

# Translation: Tendência da incompletude da variável raça/cor nas internações por covid-19 cujo desfecho foi óbito no Brasil, 2020–2022

**Authors:** Hebert Luan Pereira Campos dos Santos, Emmanuel Santos Trindade, Esly Rebeca Amaral Oliveira, Marcos Vinicius da Silva Cordeiro, Rian Silva de Oliveira, Elvira Caires de Lima, Adriano Maia dos Santos, Nília Maria de Brito Lima Prado

**Affiliations:** I Brasil Universidade Federal da Bahia. Instituto de Saúde Coletiva. Salvador, BA, Brasil; II Brasil Universidade Estadual do Sudoeste da Bahia. Vitória da Conquista, BA, Brasil; III Brasil Universidade Federal da Bahia. Instituto Multidisciplinar em Saúde. Programa de Pós-Graduação em Psicologia da Saúde. Vitória da Conquista, BA, Brasil; IV Brasil Universidade Federal do Espírito Santo. Programa de Pós-Graduação em Saúde Coletiva. Vitória, ES, Brasil; V Brasil Universidade Federal da Bahia. Instituto Multidisciplinar em Saúde. Programa de Pós-Graduação em Saúde Coletiva. Vitória da Conquista, BA, Brasil

**Keywords:** Sistemas de Informação, População Negra, Estudos de Séries Temporais, Observatórios de Saúde, COVID-19, Saúde das Minorias Étnicas, Pandemias, Desigualdades Étnicas em Saúde

## Abstract

**OBJETIVO:**

Analisar a incompletude e a tendência da incompletude da variável raça/cor nas internações por covid-19 cujo desfecho foi óbito, no Brasil, no período entre abril de 2020 e abril de 2022.

**MÉTODOS:**

Estudo ecológico de série temporal sobre a incompletude da variável raça/cor nas internações por covid-19 cujo desfecho foi óbito no Brasil, suas macrorregiões e Unidades Federativas (UF), pela regressão por *joinpoint*, cálculo da *Monthly Percent Change* (MPC) e *Average Monthly Percent Change* (AMPC), a partir de dados do Sistema de Informação Hospitalar do Sistema Único de Saúde (SIH/SUS).

**RESULTADOS:**

A incompletude da variável raça/cor nas internações por covid-19 cujo desfecho foi óbito no Brasil foi 25,85%, considerada ruim. Todas as regiões do país tiveram grau de incompletude ruim, exceto a Região Sul, considerada regular. No período analisado, a análise *jointpoint* revelou tendência de estabilidade na incompletude da variável raça/cor no Brasil (AMPC = 0,54; IC95% -0,64 a 1,74; p = 0,37) e nas regiões Sudeste (AMPC = -0,61; IC95% -3,36 a 2,22; p = 0,67) e Norte (AMPC = 3,74; IC95% -0,14 a 7,78; p = 0,06). As regiões Sul (AMPC = 5,49; IC95% 2,94 a 8,11; p = 0,00002) e Nordeste (AMPC = 2,50; IC95% 0,77 a 4,25;p = 0,005) apresentaram crescimento na tendência da incompletude, enquanto a Região Centro-Oeste (AMPC = -2,91; IC95% -5,26 a -0,51; p = 0,02) teve tendência de redução.

**CONCLUSÃO:**

A proporção de preenchimento ruim e a tendência de estabilidade da incompletude revelam que não houve melhoria na qualidade de preenchimento da variável raça/cor durante o período da pandemia da covid-19 no Brasil, fato que pode ter ampliado as iniquidades em saúde para população negra e dificultado o planejamento de ações estratégias para essa população, considerando o contexto pandêmico. Os resultados encontrados reforçam a necessidade de fomentar a discussão sobre o tema, tendo em vista que a incompletude dos sistemas de informação em saúde amplia desigualdades no acesso aos serviços de saúde e compromete a qualidade dos dados em saúde.

## INTRODUÇÃO

O desenvolvimento de políticas de saúde concatenadas com a realidade da população encontra-se diretamente relacionado com o acesso à informação de qualidade, sendo sua disponibilidade fundamental para a análise da situação de saúde e a tomada de decisão no âmbito das políticas e do cuidado em saúde, com vistas a reduzir as iniquidades e desigualdades em saúde^1^. No Brasil, a atual estrutura de governança de dados e informações de saúde no Sistema Único de Saúde (SUS) é considerada uma iniciativa robusta por contemplar uma gama de Sistemas de Informação em Saúde (SIS), de abrangência nacional e com grande parte dos dados disponíveis por acesso via Internet capitaneadas pelo Ministério da Saúde^2,3^.

Como esforço conjugado, ante a existência de inúmeros sistemas de informação estruturados a partir de estratégias individualizadas, atualmente tem sido proposta a integração e a interoperabilidade entre os SIS^4^ com vistas a fortalecer “o processo de planejamento, operação e controle dos serviços de saúde”^3^. Um dos pilares desse processo é a fidedignidade, que envolve indicadores de qualidade, como cobertura, grau de completude, confiabilidade e oportunidade desses sistemas, consistência e validade dos dados, fundamental para elaboração e implantação de políticas voltadas para a melhoria da saúde da população^1,5^.

Considerando-se a dimensão de completude dos dados no SUS, diversos estudos^6^brasileiros têm indicado alto grau de variação quanto à incompletude de muitas variáveis. A variável raça/cor, por exemplo, tem atingido baixo grau de completude em um conjunto de condições e doenças^9,10^. No contexto da pandemia por *coronavirus disease* 2019 (covid-19), por exemplo, estudos apontaram escassez de dados desagregados por raça/cor, o que impossibilitou a verificação fidedigna dos impactos da pandemia sobre esse público e prejudicou o desenvolvimento de planos estratégicos específicos^11^. Santos et al.^11^ apontaram que, no início da pandemia, das 27 Unidades Federativas (UF), apenas 19% divulgavam os dados desagregados por raça/cor para os casos confirmados, casos de síndrome respiratória aguda grave por covid-19 hospitalizados e óbitos por covid-19.

Ainda que o conceito biológico de raça tenha sido superado do ponto de vista teórico, a raça/cor é entendida como um constructo social que determina condições objetivas de desigualdade nas condições de vida e morte e o racismo institucionalizado nas práticas e serviços no Brasil^12^. Apesar da extensa experiência com a implantação de SIS no Brasil, da variável raça/cor ter sido inserida no Sistema de Informação Hospitalar do SUS (SIH/SUS) desde 2008, da Política Nacional de Saúde Integral da População Negra (PNSIPN)^13^orientar o preenchimento dessa variável e da obrigatoriedade definida pela Portaria nº 344 de 2017^14^, ainda se verifica grande variação no grau de incompletude.

O Painel de Indicadores do SUS^15^ já sinalizava a necessidade de preenchimento efetivo do item raça/cor nas fichas e sistemas de informação de saúde. Entretanto, são incipientes^16^os trabalhos publicados que analisam o grau de incompletude da variável raça/cor, no contexto da pandemia da covid-19, conforme dados dos sistemas de informação do SUS.

Assim, o objetivo desse estudo é analisar a incompletude e a tendência da incompletude da variável raça/cor nas internações por covid-19 cujo desfecho foi óbito, no Brasil, no período entre abril de 2020 e abril de 2022.

## MÉTODOS

Trata-se de um estudo ecológico de série temporal por meio da análise da tendência da incompletude da variável raça/cor nas internações por covid-19 cujo desfecho foi o óbito, no Brasil, suas macrorregiões e UF, no período entre abril de 2020 e abril de 2022.

### Fontes de Dados e Variáveis Selecionadas

As informações sobre mortalidade utilizadas neste trabalho são oriundas das internações hospitalares de pacientes que tiveram como desfecho óbito e como diagnóstico principal infecção por coronavírus de localização não especificada (CID B34.2) ou coronavírus, como causa de doenças classificadas em outros capítulos (CID B97.2), no período compreendido entre abril de 2020 e abril de 2022. Os microdados foram obtidos a partir do SIH/SUS do Departamento de Informática do SUS (Datasus) do Ministério da Saúde^19^. As variáveis utilizadas foram óbito por: raça/cor, região e UF.

O recorte temporal escolhido compreendeu os meses de abril 2020 e abril de 2022. A delimitação desse horizonte temporal justifica-se, pois abril de 2020 foi o mês em que a Defensoria Pública da União (DPU) ajuizou ação com intuito obrigar a coleta de dados referentes a raça/cor dos sistemas de vigilância epidemiológica para covid-19^20^ e, em abril de 2022, foi declarado o encerramento da Emergência em Saúde Pública de Importância Nacional (Espin)^21^.

O preenchimento da variável raça/cor é regido pela Portaria nº 344/2017, que estabelece que a coleta do quesito cor e o preenchimento do campo denominado raça/cor são obrigatórios aos profissionais atuantes nos serviços de saúde, de forma a respeitar o critério de autodeclaração do usuário de saúde, dentro dos padrões utilizados pelo Instituto Brasileiro de Geografia e Estatística (IBGE) que constam nos formulários dos sistemas de informações da saúde como: 1) branca; 2) preta; 3) amarela (pessoa de origem oriental: japonesa, chinesa e coreana, entre outras); 4) parda (inclui-se nessa categoria: morena, mulata, cabocla, cafuza ou qualquer outro mestiço de pessoa da cor preta com pessoa de outra cor ou raça); 5) indígena (aplica-se aos indígenas ou índios que vivem em aldeamento, como também aos que se declararam indígenas e vivem fora do aldeamento)^14^. A portaria estabelece ainda que, nos casos de recém-nascidos, óbitos ou diante de situações em que o usuário estiver impossibilitado para a autodeclaração, caberá aos familiares ou responsáveis a declaração de sua cor ou pertencimento étnico-racial e, nos casos em que não houver responsável, os profissionais de saúde que realizarem o atendimento deverão preencher o campo raça/cor^14^.

### Análise dos Dados

Foi calculado o percentual de incompletude (preenchimento em branco ou ignorado) da variável raça/cor para o Brasil, cada região geográfica (Norte, Nordeste, Sul, Centro-Oeste e Sudeste) e UF, por mês e durante todo o período. A incompletude refere-se aos campos em branco, não preenchido, ignorado ou situações em que é preenchido, porém com número inexistente/errado da variável^22^. Utilizou-se os pontos de corte definidos por Romero e Cunha^22^, amplamente utilizados na literatura para definição de incompletude: excelente, quando a variável apresenta menos de 5% de preenchimento incompleto; bom (de 5,0% a 9,9% de preenchimento incompleto); regular (de 10,0% a 19,9%); ruim (de 20,0% a 49,9%); e muito ruim (incompletude em 50,0% ou mais).

Para a análise de tendência da incompletude da variável raça/cor, empregou-se a regressão por *joinpoint*, por meio do *software Joinpoint Regression Program* versão 4.8.0.1, assumindo como variável regressora os meses dos anos, e estratificação por regiões e UF. A regressão *joinpoint* é uma forma de analisar as tendências temporais, avaliando pontos de junção (*joinpoints*) e se existem alterações no padrão dessa tendência, esse modelo ajusta uma série de linhas e seus pontos de junção em uma escala logarítmica, para demonstração das tendências^23,24^.

Foram calculadas: 1) a variação percentual mensal ou *Monthly Percent Change* (MPC) para cada segmento; e 2) a variação percentual mensal média ou *Average Monthly Percent Change* (AMPC), que é a média geométrica ponderada das diferentes MPC, com peso igual ao tamanho do segmento para cada intervalo de tempo. A seleção do número de pontos de junção para obter o modelo significativo foi realizada utilizando-se as configurações-padrão do *software*. Para o teste de significância, aplicou-se o valor de p, que ajusta a melhor linha para cada segmento. Uma vez que esses segmentos são estabelecidos, os respectivos percentuais mensais de mudança são estimados e testados. Quando há um ponto de junção em que o sentido se inverte ou são observados diferentes padrões de tendência, os períodos são analisados separadamente, caso não haja mudança, o período é analisado de forma integral^23,24^.

Foram considerados estatisticamente significativos os MPC e AMPC associados ao valor de p < 0,05. O cenário de crescimento na tendência da incompletude indicou a piora no preenchimento da variável; e a diminuição, a melhora desse cenário. A tendência, quando não significativa (valor de p ≥ 0,05), foi considerada como estacionária, ou seja, não demonstrou crescimento e tampouco redução estatisticamente significativa em sua série temporal, independentemente do valor de MPC ou AMPC.

Por utilizar exclusivamente dados anônimos e disponibilizados publicamente, conforme a Resolução nº 466, de 2012, do Conselho Nacional de Saúde, não foi necessário submeter o projeto à apreciação de um comitê de ética em pesquisa com seres humanos.

## RESULTADOS

Durante abril de 2020 a abril de 2022, foram registradas 384.704 internações por covid-19 cujo desfecho foi o óbito no Brasil, sendo 25,85% (n = 99.465) delas com preenchimento incompleto da variável raça/cor. Considerando todo o período analisado, a incompletude para a variável raça/cor no Brasil foi considerada ruim (25,85%) segundo o sistema de classificação proposto por Romero e Cunha^22^, comportamento similar ao de suas regiões, à exceção da Região Sul, onde foi considerada regular (12,94%) (Tabela 1). Das 27 UF brasileiras, a maioria (n = 11) apresentou grau de incompletude ruim, conforme Tabela 1. A Figura 1 ilustra a variação mês a mês da incompletude da variável raça/cor nas internações por covid-19 cujo desfecho foi óbito no Brasil e em suas regiões. A análise *jointpoint* evidencia que o Brasil apresentou tendência de estabilidade da incompletude da variável raça/cor nas internações por covid-19 (AMPC = 0,54; IC95% -0,64 a 1,74; p = 0,37) (Tabela 2).

**Tabela 1:** Percentual de incompletude da variável raça/cor nas internações por covid-19 cujo desfecho foi óbito por região geográfica e Unidade da Federação, Brasil, abril/2020–abril/2022.

Região/UF	Incompletude (%)	Classificação^a^
Brasil	25,85	Ruim
Região Norte	20,72	Ruim
Rondônia	34,52	Ruim
Acre	10,59	Regular
Amazonas	15,77	Regular
Roraima	6,46	Bom
Pará	21,13	Ruim
Amapá	57,52	Muito ruim
Tocantins	3,01	Excelente
Região Nordeste	39,18	Ruim
Maranhão	51,76	Muito ruim
Piauí	50,36	Muito ruim
Ceará	27,93	Ruim
Rio Grande do Norte	47,36	Ruim
Paraíba	41,81	Ruim
Pernambuco	36,78	Ruim
Alagoas	28,28	Ruim
Sergipe	75,67	Muito ruim
Bahia	32,65	Ruim
Região Centro-Oeste	40,62	Ruim
Mato Grosso do Sul	16,87	Regular
Mato Grosso	29,89	Ruim
Goiás	51,12	Muito ruim
Distrito Federal	57,28	Muito ruim
Região Sudeste	22,17	Ruim
Minas Gerais	16,6	Regular
Espírito Santo	18,27	Regular
Rio de Janeiro	38,72	Ruim
São Paulo	18,39	Regular
Região Sul	12,94	Regular
Paraná	15,24	Regular
Rio Grande do Sul	16,32	Regular
Santa Catarina	2,72	Excelente

Fonte: elaborado pelos autores a partir dos dados do Datasus.

^a^ Segundo Romero e Cunha^22^.

**Figura 1:** Percentual de incompletude da variável raça/cor nas internações por covid-19 cujo desfecho foi o óbito por região geográfica e Unidade da Federação, Brasil, abril/2020–abril/2022.

**Tabela 2:** Tendência da incompletude da variável raça/cor nas internações por covid-19 cujo desfecho foi óbito por região geográfica e Unidade da Federaçãoa, Brasil, abril/2020–abril/2022.

Região/UF	Período	MPC (IC95%)	Valor de p	Tendência	AMPC (IC95%)	Valor de p	Tendência
Brasil	abr/20–jun/20	16,96 (4,92 a 30,38)	0,008	Crescimento	0,54 (-0,64 a 1,74)	0,37	Estabilidade
	jun/20–nov/20	-4,66 (-7,89 a -1,33)	0,010	Redução			
	nov/20–ago/21	2,48 (1,27 a 3,70)	0,0006	Crescimento			
	ago/21–abr/21	-2,04 (-3,19 a -0,87)	0,002	Redução			
Região Norte	abr/20–jun/20	32,26 (-11,54 a 97,74)	0,16	Estabilidade	3,74 (-0,14 a 7,78)	0,06	Estabilidade
	jun/20–dez/21	-0,66 (-2,05 a 0,75)	0,33	Estabilidade			
	dez/21–abr/22	11,71 (-1,63 a 26,86)	0,08	Estabilidade			
Amazonas	abr/20–abr/22	-0,03 (-2,64 a 2,66)	0,98	Estabilidade	-0,03 (-2,64 a 2,66)	0,98	Estabilidade
Pará	abr/20–set/20	13,17 (-3,99 a 33,41)	0,13	Estabilidade	2,13 (-2,58 a 7,06)	0,38	Estabilidade
	set/20–ago/21	-10,31 (-15,30 a -5,02)	0,0009	Redução			
	ago/21–abr/22	14,50 (5,67 a 24,07)	0,002	Crescimento			
Região Nordeste	abr/20–jun/20	24,07 (0,54 a 53,09)	0,04	Crescimento	2,50 (0,77 a 4,25)	0,005	Crescimento
	jun/20–abr/22	0,73 (0,23 a 1,24)	0,006	Crescimento			
Maranhão	abr/20–jun/20	55,78 (-2,86 a 149,81)	0,06	Estabilidade	2,06 (-3,19 a 7,60)	0,45	Estabilidade
	jun/20–fev/22	0,47 (-0,92 a 1,89)	0,49	Estabilidade			
	fev/22–abr/22	-21,77 (-51,22 a 25,45)	0,29	Estabilidade			
Piauí	abr/20–jun/20	-31,85 (-55,81 a 5,10)	0,08	Estabilidade	-3,09 (-6,43 a 0,37)	0,08	Estabilidade
	jun/20–abr/22	0,06 (-0,96 a 1,10)	0,9	Estabilidade			
Ceará	abr/20–set/21	3,85 (1,65 a 6,09)	0,002	Crescimento	2,76 (-4,96 a 11,11)	0,49	Estabilidade
	set/21–dez/21	-28,11 (-60,93 a 32,29)	0,27	Estabilidade			
	dez/21–abr/22	28,47 (5,94 a 55,80)	0,01	Crescimento			
Rio Grande do Norte	abr/20–abr/22	1,85 (0,64 a 3,07)	0,004	Crescimento	1,85 (0,64 a 3,07)	0,004	Crescimento
Paraíba	abr/20–abr/22	-0,39 (-2,23 a 1,47)	0,66	Estabilidade	-0,39 (-2,23 a 1,47)	0,66	Estabilidade
Pernambuco	abr/20–jun/20	38,54 (-3,12 a 98,10)	0,07	Estabilidade	3,62 (-1,86 a 9,41)	0,2	Estabilidade
	jun/20–nov/20	-9,16 (-18,87 a 1,72)	0,09	Estabilidade			
	nov/20–fev/21	27,52 (-10,82 a 82,34)	0,17	Estabilidade			
	fev/21–abr/22	-0,33 (-1,99 a 1,35)	0,68	Estabilidade			
Alagoas	abr/20–ago/20	-36,29 (-57,68 a -4,08)	0,03	Redução	-4,51 (-10,95 a 2,40)	0,2	Estabilidade
	ago/20–abr/22	3,54 (-0,07 a 7,28)	0,05	Estabilidade			
Bahia	abr/20–abr/22	-1,98 (-3,49 a -0,45)	0,01	Redução	-1,98 (-3,49 a -0,45)	0,01	Redução
Região Centro-Oeste	abr/20–nov/20	-9,27 (-12,53 a -5,90)	0,00003	Redução	-2,91 (-5,26 a -0,51)	0,02	Redução
	nov/20–fev/21	7,80 (-5,98 a 23,60)	0,26	Estabilidade			
	fev/21–abr/22	-2,49 (-3,88 a -1,08)	0,002	Redução			
Goiás	abr/20–abr/22	-1,79 (-2,95 a -0,62)	0,004	Redução	-1,79 (-2,95 a -0,62)	0,004	Redução
Distrito Federal	abr/20–abr/22	-2,03 (-2,80 a -1,25)	0,00002	Redução	-2,03 (-2,80 a -1,25)	0,00002	Redução
Região Sudeste	abr/20–jun/20	12,48 (-7,30 a 36,48)	0,21	Estabilidade	-0,61 (-3,36 a 2,22)	0,67	Estabilidade
	jun/20–set/20	-10,06 (-25,88 a 9,14)	0,26	Estabilidade			
	set/20–set/21	2,47 (1,14 a 3,82)	0,001	Crescimento			
	set/21–abr/22	-4,97 (-7,40 a -2,48)	0,0008	Redução			
Rio de Janeiro	abr/20–abr/22	0,65 (-0,48 a 1,80)	0,25	Estabilidade	0,65 (-0,48 a 1,80)	0,25	Estabilidade
São Paulo	abr/20–jun/20	17,57 (-11,27 a 55,79)	0,24	Estabilidade	-1,77 (-5,74 a 2,36)	0,4	Estabilidade
	jun/20–set/20	-13,87 (-35,00 a 14,13)	0,27	Estabilidade			
	set/20–nov/21	0,90 (-0,58 a 2,40)	0,22	Estabilidade			
	nov/21–abr/22	-8,23 (-13,83 a -2,27)	0,01	Redução			
Região Sul	abr/20–jun/20	73,07 (32,04 a 126,84)	0,0005	Crescimento	5,49 (2,94 a 8,11)	0,00002	Crescimento
	jun/20–out/21	2,77 (1,60 a 3,95)	0,0001	Crescimento			
	out/21–abr/22	-4,09 (-8,38 a 0,39)	0,07	Estabilidade			
Paraná	abr/20–ago/20	31,79 (14,08 a 52,25)	0,0007	Crescimento	5,48 (2,92 a 8,11)	0,00002	Crescimento
	ago/20–abr/22	0,89 (-0,37 a 2,16)	0,16	Estabilidade			

MPC: variação percentual mensal (do inglês *monthly percent change*); IC95%: intervalo de confiança de 95%; AMPC: variação percentual mensal média (do inglês *average monthly percent change*).

^a^ Os Estados Rondônia, Acre, Roraima, Amapá, Tocantis, Minas Gerais, Sergipe, Espírito Santo, Minas Gerais, Rio Grande do Sul, Santa Catarina apresentaram ao menos um mês com incompletude igual a 0%, incompatível com o método baseado em cálculos logarítmicos, por isso foram excluídos da tabela.

Na Região Norte, a incompletude foi considerada ruim. Dos sete estados da região, dois (Rondônia e Pará) apresentaram grau de incompletude ruim e, no Amapá, foi considerado muito ruim (Tabela 1). Na análise *jointpoint*, a região (AMPC = 3,74; IC95% -0,14 a 7,78; p = 0,06) e os estados do Amazonas (AMPC = -0,03; IC95% -2,64 a 2,66; p = 0,98) e do Pará (AMPC = 2,13; IC95% -2,58 a 7,06; p = 0,38) também tenderam à estabilidade (Tabela 2).

O Nordeste apresentou grau de incompletude ruim (39,18%): seis estados foram considerados ruins (Ceará, Rio Grande do Norte, Paraíba, Pernambuco, Alagoas e Bahia) e três, muito ruins (Maranhão, Piauí e Sergipe) (Tabela 1). Na análise *jointpoint*, o Nordeste apresentou tendência de crescimento (AMPC = 2,50; IC95% 0,77 a 4,25; p = 0,005), embora a maioria dos estados nordestinos tenha demonstrado tendência de estabilidade, à exceção do Rio Grande do Norte, com tendência de crescimento (AMPC = 1,85; IC95% 0,64 a 3,07; p = 0,004), e da Bahia, com tendência de redução (AMPC = -1,98; IC95% -3,49 a -0,45; p = 0,01) (Tabela 2).

O Centro-Oeste foi a região que apresentou o maior grau de incompletude (40,62%), sendo classificado como ruim. Duas UF foram consideradas muito ruins (Goiás e Distrito Federal) (Tabela 1). Na análise da tendência da incompletude, a Região Centro-Oeste (AMPC = -2,91; IC95% -5,26 a -0,51; p = 0,02) tendeu à redução, comportamento também apresentado por Goiás (AMPC = -1,79; IC95% -2,95 a -0,62; p = 0,004) e pelo Distrito Federal (AMPC = -2,03; IC95% -2,80 a -1,25; p = 0,00002) (Tabela 2).

Na Região Sudeste o grau de incompletude foi considerado ruim (22,17%), embora a maioria dos estados (São Paulo, Espírito Santo e Minas Gerais) tenha sido considerado regular (Tabela 1). Na análise *jointpoint*, o Sudeste (AMPC = -0,61; IC95% -3,36 a 2,22; p = 0,67) e os estados do Rio de Janeiro (AMPC = 0,65; IC95% -0,48 a 1,80; p = 0,25) e de São Paulo (APMC = -1,77; IC95% -5,74 a 2,36; p = 0,40) apresentaram tendência de estabilidade (Tabela 2).

A Região Sul foi a única que apresentou grau de incompletude regular (12,94 %) e o estado de Santa Catarina foi o único do país a apresentar grau de incompletude excelente (Tabela 1). Na análise *jointpoint,* a região (AMPC = 5,49; IC95% 2,94 a 8,11; p = 0,00002) e o estado do Paraná (AMPC = 5,48; IC95% 2,92 a 8,11; p = 0,00002) apresentaram tendência de crescimento (Tabela 2).

## DISCUSSÃO

Os resultados dessa pesquisa apontam que o grau de incompletude da variável raça/cor nas internações por covid-19 cujo desfecho foi óbito, entre abril de 2020 e abril de 2022, foi ruim em todo território brasileiro. Esses dados corroboram com a literatura nacional quanto ao grau de preenchimento da variável raça/cor em diversos sistemas de informação do SUS, em diversos períodos^8^e em um conjunto de doenças^16^.

Já a série histórica revelou tendência de estabilidade do grau de incompletude no país, resultado divergente do encontrado por alguns estudos nacionais em algumas doenças e agravos^16^. Esses achados podem ser divergentes, pois em contextos de emergência em saúde pública e alta demanda dos serviços de saúde, a disponibilidade e a qualidade de preenchimento dos dados sobre raça/cor costumam ser prejudicadas^25^. Ao longo da pandemia, apesar de algumas iniciativas com vistas a aprimorar a coleta de dados, dificuldades no manejo dos sistemas de informação prevaleceram em todos os níveis de gestão, seja por preenchimento incompleto de informações, com variação da qualidade entre os órgãos e instituições responsáveis pela coleta seja pelas mudanças no processo de coleta, fluxo e instrumentos direcionadas a notificação dos casos^26^.

No contexto internacional, por exemplo, as evidências apontaram para disparidades entre grupos étnico-raciais nas taxas de hospitalização e mortalidade, no acesso a serviços de saúde durante a pandemia e na vulnerabilidade à covid-19 ter sido mascarada por rótulos raciais/étnicos, indicando deficiências das estratégias de saúde pública e necessidade de coleta sistemática dessas variáveis para a identificação de grupos de risco^27^. Sabe-se que muitos países de baixa e média renda não possuem sistemas de vigilância adequados e infraestruturas de saúde responsivas para resolver esses problemas de maneira confiável^30^. Sobre esse aspecto, Otu et al.^31^ recomendam a criação de SIS que facilitem a aquisição e a utilização de dados desagregados durante contextos pandêmicos e fora dele, enfatizando a possibilidade de identificar tendências relacionadas com a raça/cor e os determinantes socioeconômicos mais amplos, buscando instituir rápidas mudanças políticas a partir de abordagens interseccionais.

No presente estudo, a Região Sul apresentou a menor média de incompletude dos registros de raça/cor das internações por covid-19 cujo desfecho foi óbito, resultado também encontrado por Souza et al.^16^, ao avaliarem a incompletude do registro da raça/cor das doenças e agravos mais prevalentes na população negra nos SIS entre 2009 e 2018. Embora os avanços nos SIS no Brasil sejam significativos, persistem determinados problemas e dificuldades em algumas regiões do país, como Norte e Nordeste^32^. Senna^33^ destaca que isso é reflexo das desigualdades sociais e regionais existentes no país, que envolvem problemas como provimento médico em determinadas localidades e em locais de difícil acesso, dificuldades de fixação de profissionais, em especial de codificadores treinados, entre outros.

Embora o Brasil tenha se destacado na formulação e evolução do SIS na América^32^ e o percentual de incompletude do campo raça/cor no âmbito nacional tenha diminuído nos últimos 15 anos^25^, os desafios para a qualificação dos dados referentes à saúde da população negra ainda permanecem. No quesito raça/cor, diversos estudos^6^têm apontado permanência do racismo interpessoal, estrutural e institucional na coleta dessa variável por parte dos profissionais dos serviços de saúde. Geraldo et al.^34^, ao descrever e analisar a implementação da coleta da raça/cor realizado pelos profissionais do setor de registro de pacientes de um hospital universitário do município de São Paulo, durante a pandemia da covid-19, constataram que a maioria dos registros do quesito raça/cor na instituição observada é feita por heteroidentificação, mesmo a Portaria nº 344/2017^14^determinando que o preenchimento do campo deva respeitar o critério de autodeclaração do usuário de saúde, exceto em alguns cenários.

Questionar com qual raça/cor o indivíduo se identifica ainda traz desconforto para muitos profissionais da saúde^34,35^, com provável tendência a um embranquecimento na heteroidentificação. Somam-se a isso a falta de conhecimento sobre a PNSIPN, a pouca compreensão sobre a importância da coleta do quesito raça/cor para o desenvolvimento de políticas públicas por parte dos profissionais de saúde, a falta de treinamento específico para o preenchimento do quesito raça/cor e a inexistência de projetos de auditoria para monitoramento do seu preenchimento^7^. Tais aspectos devem ser considerados como barreiras importantes no processo de coleta e sistematização dos dados, visto que são eles que orientam a formulação das políticas de saúde no país.

O III Plano Operativo PNSIPN de 2017^36^ tinha como uma das ações estratégicas qualificar a coleta, o processamento, a análise e a publicação dos dados desagregados por raça/cor. Entretanto, no Brasil, os estudos sobre a pandemia da covid-19 sinalizaram para uma política explícita de invisibilização de dados que desvelam iniquidades étnico-raciais, sobretudo, pela falta de divulgação de dados desagregados por raça/cor/etnia^11,22^. Mesmo com a obrigatoriedade do preenchimento raça/cor desde 2017^14^, a variável não constou entre os boletins epidemiológicos publicados no início da pandemia no país, sendo incorporada apenas após pressão de movimentos sociais e entidades ligadas ao campo da saúde^11,36^. Araújo et al.^37^apontaram, por exemplo, que, mesmo quando o estado brasileiro passou a divulgar os dados desagregados, isso ocorreu a partir de uma baixa qualidade, fator que inviabilizou análises robustas do impacto da pandemia em minorias étnicas.

Deve-se reconhecer que a qualidade da informação sobre raça/cor é condição necessária para reconhecer o impacto das iniquidades em saúde na mortalidade e no acesso aos serviços de saúde. Há necessidade de aprimoramento do registro, da coleta e da divulgação dos dados no âmbito de epidemias como o representando pela covid-19, considerando não apenas raça/cor, mas ocupação, local de residência e outras variáveis que permitam compreender as relações entre o processo de determinação social da saúde e seu impacto nos indicadores de morbimortalidade da população brasileira^26^.

As discrepâncias regionais quanto ao grau de completude revelam desafios importantes para o Ministério da Saúde na efetivação de um SIS que contribua efetivamente para um SUS equânime e universal. Diversos fatores podem estar envolvidos nessas disparidades regionais, como: escritas ilegíveis, fluxo deficiente de informação, desconhecimento ou pouca importância por parte dos profissionais quanto ao preenchimento dos dados, entre outros^10^.

Alguns estudos^10,22^, ao analisarem os SIS brasileiros, apontaram maior grau de incompletude nos anos iniciais das análises, com melhora no decorrer dos anos analisados. Entretanto, no contexto de emergências sanitárias, SIS robustos, considerando cobertura, grau de completude, confiabilidade, consistência e validade dos dados tornam-se fundamentais para a correta resposta a estes eventos. O esforço para o desenvolvimento de políticas intersetoriais para superação das graves consequências da incompletude dos dados deve ser empreendido visando diminuir as iniquidades sociais, em especial para as populações mais vulnerabilizadas.

O presente estudo apresenta limitações relativas aos métodos, considerando que o uso de dados secundários pode levar à subestimação pela incompletude e inconsistências nas bases de origem. Tais limitações podem ser classificadas em três tipos, quais sejam: a primeira diz respeito à extração dos dados de óbitos terem sido retirados das informações consolidadas das autorizações de internação hospitalar (AIH), entretanto é o único SIH de domínio público do país que tem disponibilizado os dados desagregados por raça/cor, em acesso público, considerando os códigos atualizados da Classificação Internacional de Doenças (CID-10) para a covid-19. A segunda diz respeito à avaliação da cobertura do sistema, que abrange apenas as internações realizadas na rede pública e conveniada ao SUS, excluindo internações realizadas de forma particular e por planos de saúde. Entretanto, o SIH/SUS é uma importante base de dados relativa à morbidade hospitalar no Brasil, e consolida grande parte das internações hospitalares ocorridas no país. A terceira envolve o fato de serem analisados apenas os óbitos de pacientes internados e que possuíam AIH, excetuando-se indivíduos que foram a óbito sem serem internados.

Os resultados obtidos apontam alguns direcionamentos no contexto político-institucional: o primeiro refere-se à necessidade de fortalecer o monitoramento e a avaliação das ações propostas pela PNSIPN, visto que, mesmo após 15 anos de sua publicação, elementos centrais da política ainda permanecem sem efetivação, a exemplo da educação permanente dos trabalhadores da saúde sobre a importância do correto preenchimento do quesito raça/cor para os SIS e para efetivação da PNSIPN. O segundo diz respeito ao fortalecimento dos SIS no contexto brasileiro, pois sistemas públicos de saúde robustos requerem SIS consistentes e confiáveis. O terceiro refere-se à urgência em fortalecer a preparação e resposta às emergências em saúde pública por parte do sistema de saúde brasileiro, com vistas à elaboração de ações eficientes, resolutivas, equânimes e concatenadas com as realidades locais, com destaque para as desigualdades sociais, bem como para as diferenças de risco relacionadas às doenças, uma vez que dados atualizados podem se traduzir em melhores tomadas de decisões locais e regionais.

Os desafios colocados pela pandemia da covid-19 em países como o Brasil, marcado pelo racismo e por significativas iniquidades sociais e raciais, podem significar uma oportunidade para redefinir as políticas de saúde pública e os cuidados ofertados a populações socialmente marginalizadas, impulsionados por gestores, formuladores de políticas públicas, profissionais e pessoas comprometidas com redução das iniquidades raciais no país.
